# Synthesis, Radiolabeling and Biological Evaluation of Propylene Amine Oxime Complexes Containing Nitrotriazoles as Hypoxia Markers

**DOI:** 10.3390/molecules17066808

**Published:** 2012-06-04

**Authors:** Huafan Huang, Lei Mei, Taiwei Chu

**Affiliations:** Beijing National Laboratory for Molecular Sciences, Radiochemistry and Radiation Chemistry Key Laboratory of Fundamental Science, College of Chemistry and Molecular Engineering, Peking University, Beijing 100871, China

**Keywords:** nitrotriazole, technetium-99m, tumor, hypoxia marker, cellular accumulation

## Abstract

Two propylene amine oxime (PnAO) complexes, **1**, containing a 3-nitro-1,2,4-triazole and **2**, containing two 3-nitro-1,2,4-triazoles, were synthesized and radiolabeled with ^99m^Tc in high labeling yields. Cellular uptakes of ^99m^Tc-**1** and ^99m^Tc-**2** were tested using a S180 cells line. Under anoxic conditions, the cellular uptakes of ^99m^Tc-**1** and ^99m^Tc-**2** were 33.7 ± 0.2% and 35.0 ± 0.7% at 4 h, whereas the normoxic uptakes of the two complexes were 6.0 ± 1.6% and 4.6 ± 0.9%, respectively. Both ^99m^Tc-**1** and ^99m^Tc-**2** displayed significant anoxic/normoxic differentials. The cellular uptakes were highly dependent on oxygen and temperature. Biodistribution studies revealed that both ^99m^Tc-**1** and ^99m^Tc-**2** showed a selective localization in tumor and slow clearance from it. At 4 h, the tumor-to-muscle ratios (T/M) were 3.79 for ^99m^Tc-1 and 4.58 for ^99m^Tc-2. These results suggested that ^99m^Tc-labeled PnAO complexes ^99m^Tc-**1** and ^99m^Tc-**2** might serve as novel hypoxia markers. By introducing a second nitrotriazole redox center, the hypoxic accumulation of the marker was slightly enhanced.

## 1. Introduction

Owing to the vascular deficiencies, hypoxic cells exist in solid tumors and are resistant to radiation [1]. To improve the efficacy of radiotherapy, numerous radiosensitizers have been developed. One important strategy to develop hypoxia markers is the use of hypoxic radiosensitizers. A variety of nitroimidazole derivatives had been synthesized and evaluated as hypoxia markers [2], especially 2-nitroimidazole derivatives. For instance, the ^18^F labelled 2-nitroimidazole derivative, [^18^F]fluoro-misonidazole ([^18^F]FMISO) had been used for the imaging of tumor hypoxia [3], stroke [4] and ischemic myocardium [5]. Recently, 4- and 5-nitroimidazolyl compounds had also been developed as hypoxia markers [6,7].

Nitrotriazole is cheaper and more readily available than 2-nitroimidazole, and nitrotriazole derivatives had also been widely used as radiosensitizers [8,9,10]. Several nitrotriazole derivatives have been investigated as hypoxia markers [11,12,13,14]. The biodistribution studies of ^99m^Tc labeled cyclam *N*-2'-methoxyethyl-2-(3'-nitro-1'-triazole) acetamide (cyclam AK 2123) [11] and ^177^Lu labeled nitrotriazole derivative [12] revealed high tumor to muscle ratios. But no *in vitro* study was conducted. The compound ^99m^Tc-1-(3-1,2,4-nitrotriazole-1-yl)-propanhydroxyiminoamide (^99m^Tc-NTPA) continuously accumulated in hypoxic cells, but not in aerobic cells, and the biodistribution results indicated that ^99m^Tc-NTPA could localize in tumors [13]. Recently, 3-nitro-1,2,4-triazole derivative [^18^F]3-NTR had been synthesized and evaluated as a hypoxia marker. However, its hypoxic cellular uptake was lower than that of [^18^F]FMISO, and the biodistribution studies and the PET imaging showed that the tumor to muscle ratios of [^18^F]3-NTR were also lower than those of [^18^F]FMISO [14].

^99m^Tc is an ideal diagnostic radionuclide with versatile chemistry [15]. In most technetium labeled nitroazole derivatives, such as oxo[[3,3,9,9-tetramethyl-1-(2-nitro-1H-imidazol-1-yl)-4,8-diaza-undecane-2,10-dione dioximato] (3-)-*N*,*N'*,*N''*,*N'''*]-technetium (BMS181321) [16,17] and oxo-[[3,3,9,9-tetramethyl-5-oxa-6-(2-nitro-1*H*-imidazol-1-yl)-4,8-diazaundecane-2,10-dione dioximato] (3-)-*N*,*N'*,*N''*,*N'''*]-technetium (BRU59-21) [18], the redox center nitroazole was combined with chelating groups to coordinate with ^99m^Tc. The chemical structure of BMS181321 was characterized using ^99^TcO(PnAO-1-(2-nitroimidazole). ^99^TcO(PnAO-1-(2-nitroimidazole) was synthesized and analyzed with X-ray crystallography [16]. It had square-pyramidal geometry about the five-coordinate ^99^Tc metal core, and oxygen occupied an apical position. 

In our previous work, several ^99m^Tc-labeled PnAO complexes containing nitroimidazoles were prepared, and the effect of a second nitroimidazole redox center on the hypoxic accumulation was investigated. It was very interesting that by introducing a second 2-nitroimidazole redox center to BMS181321, the hypoxic accumulation increased significantly. However, a second 4-nitroimidazole showed little effect [19]. 

**Figure 1:**
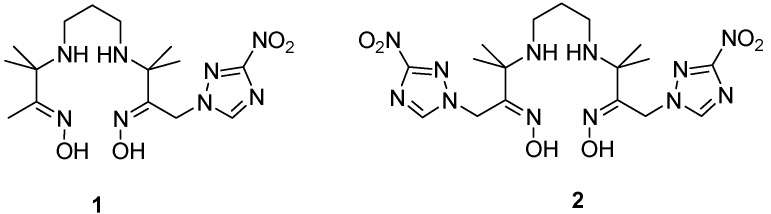
The structure of PnAO complexes **1** and **2**.

To study the effect of a second nitrotriazole, another redox center on the hypoxic accumulation, in this study, using nitrotriazole as redox center and propylene amine oxime (PnAO) as chelating group, we synthesized PnAO complexes **1**, containing a 3-nitro-1,2,4-triazole and **2**, containing two 3-nitro-1,2,4-triazoles (Figure 1). Compounds **1** and **2** were labeled with ^99m^Tc. The *in vitro* and *in vivo* evaluation was also investigated.

## 2. Results and Discussion

### 2.1. Synthesis

**Scheme 1:**
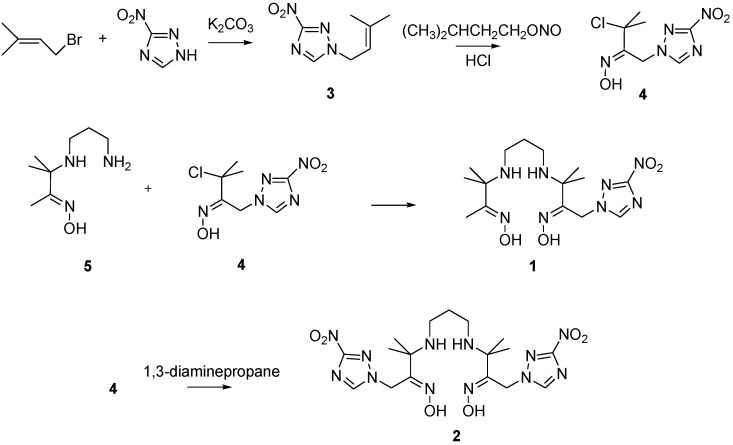
The synthesis of compound **1** and **2**.

The synthesis routes of PnAO complexes **1** and **2** are shown in Scheme 1. Alkylation of 3-nitro-1*H*-1,2,4-triazole with bromo olefin provided compound **3**. Chloronitroso derivative **4** was obtained by the addition of conc. HCl to the mixture of isoamyl nitrite and olefin **3**, and then the reaction between chloronitroso derivative **4** and diamine mono oxime **5** afforded precursor **1**. Precursor **2** was achieved via the reaction of chloronitroso derivative **4** with 1,3-diaminopropane 

### 2.2. Radiolabeling

**Figure 2:**
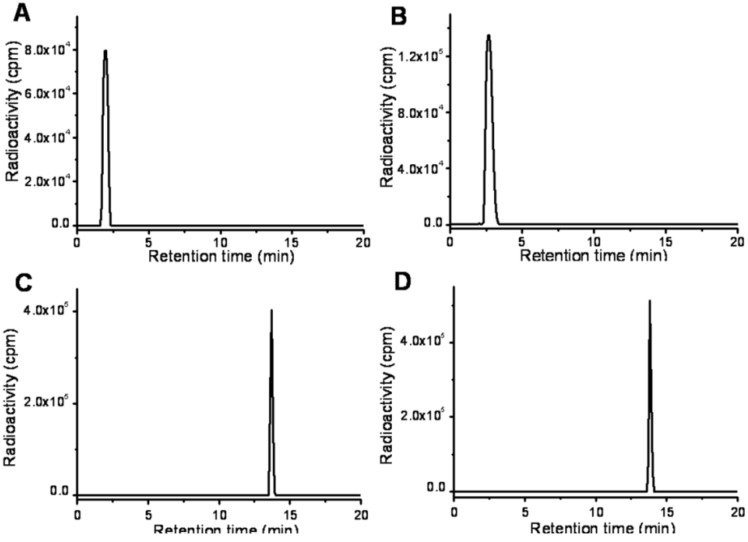
Quality control analysis of labeling mixture. Radioactivity signals of ^99m^Tc-DTPA (**A**), ^99m^TcO4^−^ (**B**), ^99m^Tc**-1** (**C**) and ^99m^Tc**-2** (**D**).

The ^99m^Tc-complexes were prepared using SnCl_2_ as a reducing agent. Precursors **1** and **2** were labeled with ^99m^Tc by ligand exchange from ^99m^Tc-DTPA. The labeling mixture was analyzed by high performance liquid chromatography (HPLC) [19]. The retention times of ^99m^Tc-DTPA and ^99m^TcO_4_^−^ were 1.8 and 2.7 min, whereas the radioactivity signals of ^99m^Tc**-1** and ^99m^Tc**-2** showed a single peak at 13.7 and 13.8 min, respectively (Figure 2). The initial radiochemical purities of ^99m^Tc**-1** and ^99m^Tc**-2** were above 95%. The radiochemical purities remained above 90% after being kept at room temperature for 8 h. The stability of ^99m^Tc**-2** containing two nitrotriazoles was better than that of the PnAO analogue containing two 2-nitroimidazoles, for which the purity remained above 80% after 4 h [19].

### 2.3. Octanol/Water Partition Coefficient

The partition coefficients were determined by the reported procedure [20,21]. The partition coefficients (log *P*_o/w_) of ^99m^Tc**-1** and ^99m^Tc**-2** were 1.74 and 1.42, respectively. ^99m^Tc**-1** was more lipophilic than ^99m^Tc**-2**.

### 2.4. *In Vitro* Study

**Figure 3:**
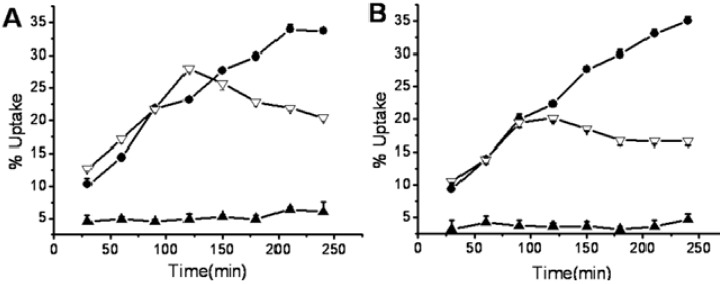
The accumulation of ^99m^Tc**-1** (**A**) and ^99m^Tc**-2** (**B**) in S180 cells under anoxic conditions (black dot), normoxic conditions (black triangle) and anoxic switched to normoxic after 2 h (blank triangle).

After labeling, *in vitro* studies of ^99m^Tc**-1** and **^9^**^9m^Tc**-2** were carried out using S180 cells. The cellular uptakes of ^99m^Tc**-1** and ^99m^Tc**-2** under normoxic and anoxic conditions were plotted versus time in Figure 3. Under normoxic conditions, the cellular uptakes of ^99m^Tc**-1** and ^99m^Tc**-2** fluctuated at a range of 3–7%. In contrast, the cellular uptakes under anoxic conditions showed a continued increase with time. Significant differences between normoxic and anoxic uptakes were observed in both ^99m^Tc**-1** and ^99m^Tc**-2** (independent t-test, *p* values <0.01). For ^99m^Tc**-1** containing one 3-nitro-1,2,4-triazole, the initial cellular uptake under anoxic condition was 10.3 ± 0.9% at 30 min, and it reached up to 14.4 ± 0.5%, 23.2 ± 0.5% and 33.7 ± 0.2% at 1, 2 and 4 h. Whereas the uptakes under normoxic condition were 4.5 ± 0.9% at 30 min, and there was little significant increase over four hours. For ^99m^Tc**-2** containing two 3-nitro-1,2,4-triazoles, the anoxic cellular uptakes were 9.4 ± 0.7%, 13.9 ± 0.3%, 22.3 ± 0.1% and 35.0 ± 0.7% at 30 min, 1, 2 and 4 h. In contrast, the normoxic uptakes were 3.1 ± 1.4% by 30 min, and the value was not statistically different during the four hours.

BMS181321 was found to selectively accumulate in anoxic CHO cells [17]. In our previous work [19], BMS181321 also accumulated in S180 cells under anoxic conditions, and the anoxic uptake was 24.4 ± 0.7% at 4 h. In the cells, nitroazoles including nitroimidazole and nitrotriazole undergo a single electron reduction catalyzed by xanthine oxidases. In the presence of sufficient oxygen, the free radical anion can be reoxidized by oxygen, and diffuse out of the cells. However, under hypoxic condition, it can be further reduced and bind to cellular macromolecules [22]. By changing 2-nitroimidazole to 3-nitro-1,2,4-triazole, the anoxic uptake increased to 33.7 ± 0.2% at 4 h. The reduction potential of 3-nitro-1, 2,4-triazole (−0.55 V) is more negative than 2-nitroimidazole (−0.40 V) [23]. Thus 2-nitroimidazole was believed to be a more efficient electron acceptor, and it can accept electrons more easily to form the radical anion. However, ^99m^Tc**-1** containing a 3-nitro-1,2,4-triazole showed higher anoxic cellular accumulation compared with BMS181321 containing a 2-nitroimidazole [19].

Both ^99m^Tc**-1** and ^99m^Tc**-2** containing nitrotriazoles showed significant anoxic/normoxic differentials. The uptake ratios (anoxic/normoxic) at 30 min, 1, 2 and 4 h were 2.3, 3.0, 4.7 and 5.6 for ^99m^Tc**-1**, and 3.0, 3.3, 6.2 and 7.5 for ^99m^Tc**-2**. ^99m^Tc**-2** showed higher anoxic/normoxic uptake ratios compared with ^99m^Tc**-1**, indicating that the anoxic/normoxic differentials increased by introducing a second 3-nitro-1, 2,4-triazole moiety into the molecule. Both ^99m^Tc**-1** and ^99m^Tc**-2** exhibited higher anoxic/normoxic uptake ratios compared with BMS181321 [19]. Thus 3-nitro-1,2,4-triazole moiety can work as a redox center with appropriate reduction potential.

In another series of cellular uptake studies, the anoxic exposure was switched to the normoxic exposure at 2 h. The cellular uptakes of both ^99m^Tc**-1** and ^99m^Tc**-2** declined slightly in the next two hours (Figure 3). Oxygen can reoxidize the free radical anion to the original drug. The entry of oxygen restrained the cellular uptakes of nitrotriazole derivatives. Therefore, there was no further accumulation after 2 h. All the metabolism products of markers would be bound to the cellular components irreversibly under ideal conditions. However, incomplete retention of metabolized markers had been reported in previous studies. For instance, BMS181321 showed 60% of the counts were retained after washing, and these counts were continually lost in the later incubation [17]. The radiolabeled metabolites of BRU59-21 appeared in the supernatants in hypoxic cells [18]. In this study, the supernatants were also analyzed with radio-HPLC during the cellular studies. The initial radiochemical purity of ^99m^Tc**-1** was above 95%. After 4 h, there was little change in the radio chromatographic signals under normoxic conditions, the radiochemical purity remained 94%. In contrast, after 4 h of incubation under anoxic condition, the radiochemical purity of ^99m^Tc**-1** was only 44%. The main metabolites showed the same retention time as ^99m^TcO4^−^. These results suggested that the metabolites of ^99m^Tc**-1** might be released from the anoxic cells into the medium. Therefore, the cellular uptakes of ^99m^Tc**-1** and ^99m^Tc**-2** declined slightly after switching to the normoxic exposure.

**Figure 4:**
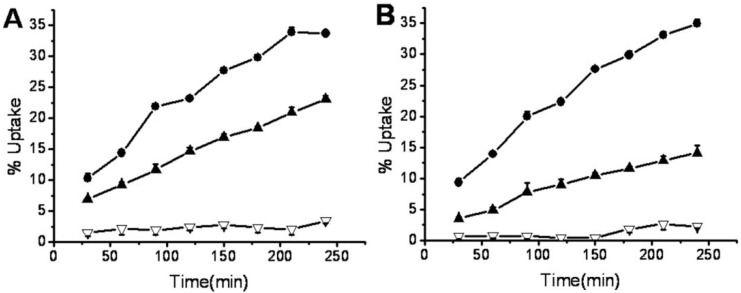
The anoxic uptakes of ^99m^Tc**-1** (**A**) and ^99m^Tc**-2** (**B**) in S180 cells at 4 °C (blank triangle), 25 °C (black triangle) and 37 °C (black dot).

To study the effect of temperature, the cellular uptake studies were also performed at 4 °C and 25 °C (Figure 4). Under anoxic conditions, the cellular uptakes of ^99m^Tc**-1** and ^99m^Tc**-2** were increased dramatically by rising the temperature. At 4 h, the anoxic uptakes of ^99m^Tc**-1** at 4 °C, 25 °C and 37 °C were 3.5 ± 0.4%, 23.1 ± 0.6% and 33.7 ± 0.2%, respectively. In contrast, the anoxic uptakes of ^99m^Tc**-2** at 4 °C, 25 °C and 37 °C were 2.3 ± 0.3%, 14.1 ± 1.2% and 35.0 ± 0.7% at 4 h. The anoxic uptakes of ^99m^Tc**-1** and ^99m^Tc**-2** strongly depended on temperature. Under normoxic conditions, the cellular uptakes were low and the measurement errors were relatively high, so the normoxic uptakes showed no significant difference at various temperatures. The differences in anoxic accumulation at 4 °C, 25 °C and 37 °C might be due to the enzyme activity and membrane fluidity. The reduction of nitrotriazole in the cell is an enzymatic process. At low temperature, enzymes (xanthine oxidases) are inactive. Enzyme activity increases gradually with rising temperature until the optimum temperature (35–45 °C for xanthine oxidases). On the other hand, the markers enter the cells by diffusion. High temperatures increase the fluidity of membranes, and the markers enter the cells more readily.

Both of ^99m^Tc**-1** (log *P*_o/w_ = 1.74) and ^99m^Tc**-2** (log *P*_o/w_ = 1.42) showed lipophilic nature, and the high partition coefficients allowed them cross the membrane readily by diffusion. ^99m^Tc**-1** was more lipophilic than ^99m^Tc**-2**, however, ^99m^Tc**-2** exhibited higher anoxic/normoxic uptake ratios. These results indicated that the lipophilicity may be an important factor in cellular accumulation, but other factors are of greater importance in this study.

### 2.5. Biodistribution Study

The *in vivo* studies were performed in Kunming mice bearing S180 tumor to determine the tumor specificity. The biodistribution results are tabulated in Table 1 and Table 2. For both ^99m^Tc**-1** and ^99m^Tc**-2**, the liver was the tissue with highest radioactivity, and the radioactivity in the kidneys was low. These results suggested lipophilic complexes ^99m^Tc**-1** and ^99m^Tc**-2** were largely cleared through the hepatobiliary pathway. The tumor uptakes decreased slowly after injection, from 0.47 ± 0.08 %ID/g at 0.5 h to 0.35 ± 0.03 %ID/g at 4 h for ^99m^Tc**-1** and 0.39 ± 0.06 %ID/g at 0.5 h to 0.25 ± 0.06 %ID/g at 4 h for ^99m^Tc**-2**. The tumor-to-blood ratios (T/B) were 0.39 for ^99m^Tc**-1** and 0.31 for ^99m^Tc**-2** at 4 h. Both ^99m^Tc**-1** and ^99m^Tc**-2** showed a slow blood clearance, similar to that observed in BMS181321 [17]. This might be due to the high octanol/water partition coefficients of these complexes. However, the uptakes in muscle were low and cleared relatively quickly. The muscle uptakes decreased from 0.19 ± 0.03 %ID/g at 0.5 h to 0.09 ± 0.01 %ID/g at 4 h for ^99m^Tc**-1**, and from 0.22 ± 0.04 %ID/g at 0.5 h to 0.05 ± 0.01 %ID/g at 4 h for ^99m^Tc**-2**. The tumor-to-muscle ratios (T/M) increased with time after injection, and the ratios were 3.79 for ^99m^Tc**-1** and 4.58 for ^99m^Tc**-2** at 4 h. Compared with BMS181321 at 4 h after injection (3.53 in KHT tumor, 4.21 in SCC tumor and 3.54 in RIF-1 tumor) [17], ^99m^Tc**-1** and ^99m^Tc**-2** showed similar or higher tumor specificity. By introducing a second nitrotriazole, ^99m^Tc**-2** was a better tumor hypoxia marker compared with ^99m^Tc**-1***.*

**Table 1:** Biodistribution of ^99m^Tc-**1** in mice bearing S180 tumour (%ID/g).

Tissue	0.5 h	1 h	2 h	4 h
Blood	2.61 ± 0.65	1.97 ± 0.72	1.32 ± 0.40	0.91 ± 0.37
Heart	0.57 ± 0.13	0.43 ± 0.04	0.28 ± 0.02	0.24 ± 0.03
Lung	1.91 ± 0.74	1.30 ± 0.12	1.21 ± 0.23	0.83 ± 0.12
Liver	12.26 ± 5.14	8.15 ± 0.59	7.41 ± 2.03	4.91 ± 0.55
Spleen	0.52 ± 0.05	0.43 ± 0.05	0.37 ± 0.03	0.30 ± 0.05
Stomach	1.47 ± 0.36	1.42 ± 0.27	1.51 ± 0.19	1.16 ± 0.18
Kidney	1.73 ± 0.42	1.41 ± 0.42	1.17 ± 0.13	0.89 ± 0.16
Muscle	0.19 ± 0.03	0.16 ± 0.05	0.11 ± 0.02	0.09 ± 0.01
Brain	0.08 ± 0.01	0.05 ± 0.01	0.05 ± 0.01	0.05 ± 0.01
Tumor	0.47 ± 0.08	0.45 ± 0.01	0.42 ± 0.05	0.35 ± 0.03
T/B	0.16 ± 0.03	0.26 ± 0.10	0.34 ± 0.12	0.39 ± 0.19
T/M	2.46 ± 0.29	2.72 ± 0.55	3.88 ± 0.40	3.79 ± 0.59

**Table 2:** Biodistribution of ^99m^Tc-**2** in mice bearing S180 tumour (%ID/g).

Tissue	0.5 h	1 h	2 h	4 h
Blood	1.30 ± 0.14	1.17 ± 0.11	0.75 ± 0.38	0.84 ± 0.25
Heart	0.38 ± 0.13	0.29 ± 0.04	0.24 ± 0.08	0.18 ± 0.03
Lung	0.57 ± 0.06	0.49 ± 0.14	0.41 ± 0.09	0.31 ± 0.03
Liver	15.90 ± 2.06	18.77 ± 3.03	13.80 ± 3.58	9.09 ± 2.40
Spleen	0.31 ± 0.02	0.32 ± 0.09	0.29 ± 0.09	0.15 ± 0.02
Stomach	2.30 ± 1.42	1.54 ± 0.30	1.00 ± 0.11	0.75 ± 0.27
Kidney	1.85 ± 0.16	1.58 ± 0.28	1.42 ± 0.15	0.92 ± 0.15
Muscle	0.22 ± 0.04	0.12 ± 0.01	0.08 ± 0.02	0.05 ± 0.01
Brain	0.03 ± 0.01	0.03 ± 0.00	0.02 ± 0.00	0.02 ± 0.00
Tumor	0.39 ± 0.06	0.34 ± 0.08	0.27 ± 0.06	0.25 ± 0.06
T/B	0.31 ± 0.03	0.30 ± 0.04	0.47 ± 0.29	0.31 ± 0.08
T/M	1.80 ± 0.39	2.87 ± 0.91	3.35 ± 0.60	4.58 ± 0.76

## 3. Experimental

### 3.1. General

3-Nitro-1H-1,2,4-triazole was purchased from Tokyo Chemical Industry Co., Ltd (Tokyo, Japan). 1,3-Diaminopropane (98%) and *N*,*N*-diisopropylethylamine (98%) were supplied from Acros Organics (Geel,Belgium). All other reagents were of analytical grade. NMR spectra were obtained on Bruker (400 MHz and 500 MHz) spectrometers (Bruker, Faellanden, Switzerland). Chemical shifts are given in ppm relative to tetramethylsilane used as an internal standard, the coupling constants are expressed in hertz, and the splitting patterns are designated as s (singlet), d (doublet), t (triplet) and m (multiplet). Mass spectra were measured on a Bruker APEX IV FTMS, positive mode, ESI. Elemental analyses were carried out on an Elementar Vario MICRO CUBE (Hanau, Germany). RP-HPLC analyses were performed on a Waters 1525 binary HPLC pump and a Waters 2487 UV absorbance dual λ detector (Milford, MA USA). The elution was also monitored with a Packard 500 TR flow scintillation radioactivity detector (Meriden, CT, USA). Murine sarcoma S180 cell line was provided by the College of Life Sciences, Peking University. Dulbecco’s Modified Eagle’s Medium (DMEM) was from Gibco BRL Life Technologies (Grand Island, NY, USA) and fetal bovine serum from Hyclone (Logan, UT, USA). The JPSJ-605 dissolved oxygen meter was purchased from REX Instrument Factory of Shanghai Precision & Scientific Instrument Co., LTD. (Shanghai, China).

### 3.2. Synthesis

The synthesis routes for compound **1** and **2** are depicted in Scheme 1. Compound **5** was synthesized according to the method reported in the literature [24].

*1-(3-Methylbut-2-enyl)-3-nitro-1H-1,2,4-triazole* (**3**). 1-Bromo-3-methylbut-2-ene (1.5 mL, 13 mmol) was added to a mixture of K_2_CO_3_ (0.72 g, 5.2 mmol) and 3-nitro-1*H*-1,2,4-triazole (0.51 g, 4.5 mmol) in acetone (15 mL). The mixture was refluxed over night. Acetone was evaporated under reduced pressure and the residue was redissolved in EtOAc. The organic layer was washed with water (3 × 20 mL) and dried by Na_2_SO_4_. Evaporation of solvent gave brown oil. The crude product was purified by column chromatography (silica gel, petroleum ether–EtOAc = 2:1) to provide **3** as a light yellow oil (0.70 g, yield 85%). ^1^H-NMR (CDCl_3_, 400 MHz) δ 8.16 (s, 1H, triazole-*H*), 5.47 (t, 1H, =C*H*CH_2_-), 4.88 (d, 2H, =CHC*H_2_*-), 1.86 (s, 3H, =C(C*H_3_*)_2_), 1.82 (s, 3H, =C(C*H_3_*)_2_); ^13^C-NMR (DMSO-d_6_, 125 MHz) δ 162.0, 146.0, 139.4, 116.8, 48.3, 25.1, 17.8; HRMS (ESI): calculated for C_7_H_10_N_4_NaO_2_ [M+Na]^+^ 205.06960, found 205.06923.

*3-Chloro-3-methyl-1-(3-nitro-1H-1,2,4-triazol-1-yl)butan-2-one oxime* (**4**). Compound **3** (0.98 g, 5.4 mmol) and isoamyl nitrite (1.6 mL, 12 mmol) was cooled to −30 °C in an acetone-dry ice bath and vigorously stirred. Concentrated hydrochloric acid (1.4 mL) was added dropwise over 30 min. The mixture was stirred at −30 °C to −20 °C for another 2 h. The precipitated was isolated by filtration and washed with cold methanol-ether = 1:5 (10 mL) to give **4** as a white solid (1.09 g, yield 82 %). ^1^H-NMR (DMSO-d_6_, 400 MHz) δ 11.97(s, 1H, C=N-O*H*), 8.88 (s, 1H, triazole-*H*), 5.33 (s, 2H, NC*H_2_*-), 1.87 (s, 6H, -C(C*H_3_*)_2_-); HRMS (ESI): calculated for C_7_H_11_ClN_5_O_3_ [M+H]^+^ 248.05449, found 248.05444.

*3-(3-(3-(Hydroxyimino)-2-methylbutan-2-ylamino)propylamino)-3-methyl-1-(3-nitro-1H-1,2,4-triazol-1-yl)butan-2-one oxime* (**1**). Compound **4** (0.054 g, 0.22 mmol), compound **5** (0.043 g, 0.25 mmol) and (*i*-Pr)_2_NEt (100 μL) were added in dry acetonitrile (2 mL), and the mixture was stirred at room temperature for 24 h. Acetonitrile was removed on a rotary evaporator. The crude product was purified by column chromatography (silica gel, CH_2_Cl_2_-CH_3_OH = 10:1) and recrystallized from CH_2_Cl_2_to provide a yellow solid (0.072 g, yield 86%), m. p. 63.5–64.3 °C; ^1^H-NMR(DMSO-d_6_, 400 MHz) δ 11.32 (s, 1H, C=N-O*H*), 10.35 (s, 1H, C=N-O*H*), 8.79 (s, 1H, triazole-*H*), 5.16 (s, 2H, NC*H*_2_), 2.23 (m, 4H, -NHC*H*_2_-), 1.69 (s, 3H, N=CC*H*_3_), 1.40 (m, 2H, -CH_2_C*H*_2_CH_2_-), 1.23 (s, 6H, C(C*H_3_*)_2_), 1.11 (s, 6H, C(C*H_3_*)_2_); ^13^C-NMR (DMSO-d_6_, 125 MHz) δ 161.4, 160.1, 155.7, 147.9, 56.7, 56.6, 43.1, 41.1, 31.3, 25.6, 25.4, 8.9; HRMS (ESI): calculated for C_15_H_29_N_8_O_4_ [M+H]^+^ 385.23063, found 385.23008.

3-(3-(3-(Hydroxyimino)-2-methyl-4-(3-nitro-1H-1,2,4-triazol-1-yl)butan-2-ylamino)propylamino)-3-methyl-1-(3-nitro-1H-1,2,4-triazol-1-yl)butan-2-one oxime (**2**). Compound **4** (0.046 g, 0.19 mmol), 1,3-diaminopropane (8 μL, 0.096 mmol) and (i-Pr)_2_NEt (50 μL) were added in dry acetonitrile (2 mL), and the mixture was stirred at 40 °C for 0.5 h. Acetonitrile was removed. The crude product was purified by column chromatography (silica gel, CH_2_Cl_2_–CH_3_OH = 10:1) and recrystallized from CH_2_Cl_2 _to provide a white solid (0.039 g, yield 84%), m.p. 158.2–159.1 °C; ^1^H-NMR (DMSO-d_6_, 400 MHz) δ 11.32 (s, 2H, C=N-OH), 8.79 (s, 2H, triazole-H), 5.16 (s, 4H, NCH_2_), 2.25 (t, 4H, -NHCH_2_-), 1.40 (m, 2H, -CH_2_CH_2_CH_2_-), 1.23 (s, 12H, C(CH_3_)_2_), 0.98 (t, 2H, -NHCH_2_CH_2_CH_2_-). ^13^C-NMR (DMSO-d_6_, 125 MHz) δ 161.4, 155.7, 147.8, 56.7, 43.1, 40.9, 31.3, 25.4; HRMS (ESI): calculated for C_17_H_29_N_12_O_6_ [M+H]^+^ 497.23275, found 497.23254. 

### 3.3. Radiolabeling

Na^99m^TcO_4_ was obtained from a ^99^Mo/^99m^Tc generator. The labeling of compound **1** and **2** was conducted according to the literature methods [17] with minor modification. ^99m^TcO4^−^ (100 µL, 15.0 MBq), the ligand solution (100 µL, 2 mg/mL), phosphate buffer solution (400 µL, pH 7.2, 0.2 mol/L), saline (400 µL), diethylenetriaminepentaacetic acid (DTPA) (2 µL, 2.5 mg/mL) were mixed in a 2 mL centrifugal tube. The mixture was purged with argon and fresh SnCl_2_ solution (1.5 µL, 1 mg/mL) was added. The labeling mixture was stirred in ice water bath for 15 min and filtrated by 0.22 µm filter. The radio high-performance liquid chromatography (radio-HPLC) method had been described in previous work [19]. The stabilities of ^99m^Tc**-1** and ^99m^Tc**-2** were investigated in saline for 8 h at 37 °C.

### 3.4. Octanol/Water Partition Coefficient

The partition coefficients were determined by the reported procedure [20,21] with minor differences. Octanol (1.0 mL), ^99m^Tc-labeling solution (0.5 mL) and saline (0.5 mL) were mixed in a 2 mL centrifugal tube. The tube was vigorously vortexed for 5 min and centrifuged at 3000 rpm for another 5 min. Five samples (10 µL) of each phase were removed into gamma counter tubes and counted for radioactivity. The *P*_O/W_ was the ratio of the radioactivity of the octanol layer to that of the water layer. Then the octanol layer (0.8 mL) was moved to another centrifugal tube, in which octanol (0.2 mL) and saline (1.0 mL) were added. The tube was vortexed and centrifuged. Samples of each phase were removed and counted. This process was repeated for several times until consistent *P*_O/W_ was obtained. 

### 3.5. *In Vitro* Study

The cellular accumulation experiments were performed according to the literature methods [25,26]. Murine sarcoma S180 cells were suspended in a fresh DMEM medium plus 10% (v/v) of fetal bovine serum (FBS). The final cell concentration was 2 × 10^6^ cells/mL. Aliquots of 20 mL were added to glass vials and gently stirred with magnetic spinbars. The glass vials were placed in water baths to control the temperature. The cells were incubated under normoxic conditions (95% air plus 5% carbon dioxide) or anoxic conditions (95% nitrogen plus 5% carbon dioxide, below 10 ppm O_2_). After an equilibration for 30–45 min, The JPSJ-605 dissolved oxygen meter read 0.00 mg/L, and the ^99m^Tc-labeling marker (5 MBq in 0.5 mL) was added to each glass vial and the final concentration of unlabeled drug was 5 µg/mL. More than 1 mL sample was removed each 30 minutes. Five aliquots (200 µL) were pipetted from each sample removed and centrifuged at 1500 rpm for 5 minutes. 180 µL of supernatant was removed for counting (A), and the cells and medium left were also counted (B). The cellular uptakes were calculated as % Uptake = [(B − A/9) / (A + B)] × 100%. The trypan blue exclusion assay revealed that S180 cells maintained more than 90% viability for 4 h.

To imitate the physiological circumstances, the temperature in the accumulation experiments was 37 °C. At 1, 2 and 4 h, aliquots of supernatant were filtrated by 0.22 µm filter. Filtered supernatant was analyzed with radio-HPLC [18]. To investigate the effect of entry of oxygen to the cellular uptakes of hypoxia markers, the anoxic exposure was switch to normoxic exposure in another series of cellular accumulation experiments. To study the effort of temperature, the cellular accumulation experiments were also conducted at 25 °C (room temperature) and 4 °C [27].

### 3.6. Biodistribution Study

All the animal experiments were performed in accordance with the national laws related to the conduct of animal experimentation. The biodistribution of ^99m^Tc-**1** and ^99m^Tc-**2** was evaluated in Kunming male mice (20–25 g) bearing the S180 tumor in the left front leg (diameter of 10–15 mm). The ^99m^Tc-complex was (1 MBq in 0.1 mL) administered by tail vein injection. The mice were sacrificed by cervical dislocation at different times after injection. Various organs and tissues were excised, weighed and counted. The percent injected dose per gram (%ID/g) of each organ/tissue or tumor was calculated by the above data. The final results were expressed as means ± SD. of five parallel experiments.

## 4. Conclusions

Two propylene amine oxime derivatives **1** and **2** containing 3-nitro-1,2,4-triazole moieties were synthesized and labeled with ^99m^Tc at high labeling efficiencies (95%). Both ^99m^Tc**-1** and ^99m^Tc**-2** were stable at 8 h after labeling. *In vitro* cellular uptake studies, ^99m^Tc**-1** and ^99m^Tc**-2** displayed significant anoxic/normoxic differentials. The anoxic cellular uptakes of ^99m^Tc**-1** and ^99m^Tc**-2** reached to 33.7 ± 0.8% and 35.0 ± 0.7% at 4 h, whereas the normoxic cellular uptakes were 6.0 ± 1.6% and 4.6 ± 0.9%, respectively. The entry of oxygen restrained the cellular uptakes of ^99m^Tc**-1** and ^99m^Tc**-2**, and the anoxic cellular uptakes were highly dependent on the temperature. In biodistribution studies, the tumor-to-muscle ratios (T/M) of ^99m^Tc**-1** and ^99m^Tc**-2** increased with time. At 4 h, the tumor-to-muscle ratios (T/M) were 3.79 for ^99m^Tc-1 and 4.58 for ^99m^Tc-2. These results suggested that nitrotriazole derivatives ^99m^Tc**-1** and ^99m^Tc**-2** might serve as novel hypoxia markers. Moreover, by introducing a second nitrotriazole redox center, ^99m^Tc**-2** showed a higher selective localization in tumor hypoxia compared with ^99m^Tc**-1**.
